# Assessing the Quality of Reporting to China’s National TB Surveillance Systems

**DOI:** 10.3390/ijerph18052264

**Published:** 2021-02-25

**Authors:** Tao Li, Lijia Yang, Sarah E. Smith-Jeffcoat, Alice Wang, Hui Guo, Wei Chen, Xin Du, Hui Zhang

**Affiliations:** 1 National Center for TB Control and Prevention, Chinese Center for Disease Control and Prevention, Beijing 102206, China; litao1@chinacdc.cn (T.L.); eliza_yang@163.com (L.Y.); chenwei@chinacdc.cn (W.C.); 2 Division of Global HIV and Tuberculosis, US Centers for Disease Control and Prevention, Atlanta, GA 30333, USA; uyi7@cdc.gov (S.E.S.-J.); ilm1@cdc.gov (A.W.); 3 Beijing Office, US Centers for Disease Control and Prevention, Beijing 100600, China; Hguo28@outlook.com

**Keywords:** tuberculosis, surveillance, inventory study, under-reporting, accuracy

## Abstract

(1) Background: The reliability of disease surveillance may be restricted by sensitivity or ability to capture all disease. Objective: To quantify under-reporting and concordance of recording persons with tuberculosis (TB) in national TB surveillance systems: the Infectious Disease Reporting System (IDRS) and Tuberculosis Information Management System (TBIMS). (2) Methods: This retrospective review includes 4698 patients identified in 2016 in China. County staff linked TB patients identified from facility-specific health and laboratory information systems with records in IDRS and TBIMS. Under-reporting was calculated, and timeliness, concordance, accuracy, and completeness were analyzed. Multivariable logistic regression was used to examine factors associated with under-reporting. (3) Results: We found that 505 (10.7%) patients were missing within IDRS and 1451 (30.9%) patients were missing within TBIMS. Of 171 patient records reviewed in IDRS and 170 patient records in TBIMS, 12.3% and 6.5% were found to be untimely, and 10.7% and 7.1% were found to have an inconsistent home address. The risk of under-reporting to both IDRS and TBIMS was greatest at tertiary health facilities and among non-residents; the risk of under-reporting to TBIMS was greatest with patients aged 65 or older and with extrapulmonary TB (EPTB). (4) Conclusions: It is important to improve the reporting and recording of TB patients. Local TB programs that focus on training, and mentoring high-burden hospitals, facilities that cater to EPTB, and migrant patients may improve reporting and recording.

## 1. Background

Disease surveillance data are a vital source of information for national and international policymakers, and can be used to evaluate the burden and time trends of diseases [1]. However, the reliability of disease surveillance may be limited by sensitivity, or the ability of the system to capture all persons with disease. Many national disease surveillance systems may provide an incomplete picture due to under-reporting diseases of public health importance [2,3,4,5,6].

Tuberculosis (TB) is a disease that has existed for millennia in the world [7]. TB is the leading cause of death from a single infectious disease [8]. The World Health Organization (WHO) indicated that 7 million persons with TB were reported globally in 2018, representing 70% of the estimated 10 million new persons with TB [8]. Thus, 30% of new persons with TB were unknown to national authorities [8]. The gap is mainly due to a combination of under-diagnosis (persons with TB are not diagnosed with TB) and under-reporting (persons with TB are diagnosed with TB, but not reported in the surveillance system). Under-diagnosis can result in anti-TB treatment initiation delay, which can lead to increased morbidity and mortality and increased duration of TB transmission [9]. Under-reporting can lead to a misinterpretation of TB disease burden. Consequently, resources may not be allocated appropriately.

Although the number of new persons with TB has significantly decreased since 2009 [10], China still suffers a high TB burden. In 2018, the WHO estimated that 866,000 people developed TB disease in China, representing 9% of all new persons with TB worldwide and placing China second in absolute number of persons with TB in the world [8]. In China, pulmonary tuberculosis (PTB) patients (both clinically diagnosed and laboratory-confirmed) should be reported to the web-based Infectious Disease Reporting System (IDRS) and recorded in the Tuberculosis Information Management System (TBIMS). Extrapulmonary TB patients can also be reported, but unlike PTB, extrapulmonary TB is not mandatory to report. IDRS, managed by the Chinese Center for Disease Control and Prevention (CDC), contains patient information of 39 infectious diseases including PTB and monitors the epidemic conditions of these infectious diseases. In a report published in 2015, IDRS covered all provincial, prefectural, and county CDCs, 98% of medical institutions at or above the county administrative level, and 94% of community medical institutions [11]. TBIMS is the surveillance system for the national TB program (NTP) and is used for managing the full course of treatment of PTB patients; it does not capture information on TB presumptive patients [12]. TBIMS is accessible by all government-appointed TB-designated facilities, which could be local CDCs, TB dispensaries, or TB-designated hospitals [12]. These two systems are independent, but there is a function within the systems to link information about patients diagnosed with TB. In 2018, the WHO estimated that 71,000 (8.2%) TB patients in China were unknown to the NTP [8].

Multiple countries, including Kenya, Spain, Korea, Cape Verde, and Egypt, have conducted inventory studies to assess TB under-reporting [13,14,15,16,17]. In 2015, China implemented a study in nine counties to identify TB under-reporting to TBIMS [12]. We developed the present study to further quantify under-reporting to both IDRS and TBIMS in six other counties and to investigate the factors associated with under-reporting of patients diagnosed with TB to the two surveillance systems. Additionally, we assessed concordance and timeliness of reported patients diagnosed with TB to the two systems. This study may complement our previous study [12] and help us improve our national surveillance strategies and, furthermore, to improve TB prevention, diagnosis, and treatment services [18].

## 2. Methods

### 2.1. Study Setting

China has the largest population of any one country, with 1.39 billion people. It is divided into 34 provincial-level administrative divisions (PLADs), which are further divided into 334 prefectures and almost 3000 counties [12,19]. We assigned 31 mainland provinces into three regions: eastern region, central region, and western region. Generally, regions decrease in socioeconomic status and increase in TB notification rate as you go from east to west [20].

In China, health facilities are divided into three levels according to their workload, diagnosis and treatment capacity, and responsibilities. Primary health facilities provide prevention, medical treatment, healthcare, and rehabilitation services directly to a single community. Secondary health facilities are regional hospitals that provide comprehensive medical and health services to multiple communities. Tertiary health facilities are hospitals that provide highly specialized medical and health services. A reporting card is used by physicians to collect patient information; the reporting card may be checked by health workers in the same facility, and then this information is entered by either the physician or health worker into IDRS or TBIMS ideally within 24 h.

### 2.2. Study Design

We conducted a retrospective inventory study to quantify under-reporting and identify the sociodemographic factors and other characteristics associated with under-reporting. We matched patient records diagnosed with TB from health facilities in 2016 with TB patient records within IDRS and TBIMS. We also assessed concordance, data accuracy, and completeness of key variables within patient medical records, and timeliness, defined as recording within 24 h after diagnosis, of matched patients.

### 2.3. Sampling Method

We adopted stratified purposive sampling to select six provinces—Guangdong and Jiangsu in eastern China, Heilongjiang and Henan in central China, and Yunnan and Sichuan in western China. In each of the provinces, we selected one county with a high TB burden (more than 200 notified patients in 2016) and capacity (at least 2 local staff members in charge of NTP work) to implement the study. The six counties are Nanshan in Guangdong, Xingyang in Jiangsu, Yilan in Heilongjiang, Liyang in Henan, Simao in Yunnan, and Lu in Sichuan. Within each county, three facilities participated: the county-level TB-designated hospital, the general hospital with the greatest patient load, and one township hospital with chest x-ray capability. Designated hospitals are the largest general hospitals and the only NTP-appointed treatment centers in each county. There are not many hospitals at the county level that can diagnose TB, and according to China’s infectious disease control law, all presumptive or confirmed TB patients should be referred to designated hospitals. Additionally, to thoroughly cover diagnosed patients in selected counties, a general hospital and a township-level hospital were also included in this study to explore the missing patients. All patients diagnosed with TB were included to quantify under-reporting to IDRS and TBIMS.

To assess concordance and timeliness of reporting diagnosed TB patients to IDRS and TBIMS, we included county-level designated TB hospitals. If fewer than 30 TB patients were diagnosed at the designated TB hospital, the largest county-level general hospital was included. If more than 30 TB patients were diagnosed in the sampled health facilities during the project period, we employed a simple random sampling method to select 30 TB patients diagnosed in the sampled health facilities from IDRS and TBIMS, respectively. If not, we included all TB patients.

### 2.4. Eligibility Criteria

We included patients diagnosed with PTB, TB pleurisy, and other extra-pulmonary TB (EPTB) according to national TB diagnosis guidelines [21,22] from 1 January 2016 to 31 December 2016. Briefly, TB is categorized into the following categories: (1) primary PTB; (2) hematogenous disseminated PTB; (3) secondary PTB; (4) tuberculosis pleuritis; and (5) extrapulmonary TB. PTB patients are classified as (a) lab-confirmed; (b) clinically diagnosed; or (c) presumptive. All presumptive TB patients were excluded.

### 2.5. Data Collection

We collected TB patient medical records in health facilities, TB surveillance records in IDRS, and TB patient treatment register records in TBIMS.

From IDRS, we exported name, age, gender, national ID number, report number and date of diagnosis, and other relevant patient characteristics for all TB patients reported in 2016 into a CSV file. From TBIMS, we also exported the same variables for all reported patients in 2016 into a CSV file, along with additional register number and sputum status.

We collected medical records of diagnosed TB patients in selected health facilities. If the health facility had an electronic hospital information system or laboratory information system, we exported records with a TB diagnosis to a CSV file. If electronic data were not available, we reviewed paper medical records from outpatient departments, inpatient wards, and laboratory reports for a TB diagnosis and added these records meeting the eligibility criteria to an MS Excel^®^ (Microsoft Corporation, Redmond, WA, USA) spreadsheet. The Excel spreadsheet included the following information: name, age, gender, national ID number, home address, date of diagnosis, health facility, department of diagnosis, data source (outpatient/inpatient/laboratory), initial diagnosis, type of TB (PTB clinically diagnosed/PTB laboratory-confirmed/pleurisy or other EPTB), and result of TB laboratory examination. We also investigated whether TB patients reviewed were residents, which was defined as someone recorded in the county or who had lived in the county for more than six months, which is usually indicated via self-report in medical records and confirmed based on the type of medical insurance, since non-residents have different insurance categories.

### 2.6. Data Deduplication

A duplicate record was defined as a record with the same national ID number, name (a different character was permitted), gender, and age (+/− 2 years was allowed) as another record. Name was use as the key index to compare, and other variables were used as supplemental values. The final deduplicated database was determined by local study implementers.

### 2.7. Record Linkage

We linked the medical records dataset (from general hospitals) and health facilities dataset (from dispensaries and clinics) based on the name of the health facilities, and renamed the new dataset DX-DATASET. This was done in order to capture data on patients treated at both general hospitals and health facilities. We used a proprietary software to match deduplicated DX-DATASET and IDRS datasets by national ID number, name, age, gender, and home address. The software provides a score demonstrating the probability of a match and a health worker reviews results for a final determination of a match. A match was defined as records from the facility dataset and IDRS with the same national ID number and at least one of the same of name, age, and gender. If the national ID number of a record was missing from either the DX-DATASET or IDRS dataset, we defined a match as records with the same name, age (+/− 2 years), gender, and home address. If a record from DX-DATASET did not match with a record in IDRS, the patient was defined as under-reported in IDRS. We used the same variables and applied the same definitions to match records from TBIMS with DX-DATASET. If a record in the DX-DATASET was not matched to a record in TBIMS, the record was defined as under-reported in TBIMS.

### 2.8. Data Analysis

We used descriptive statistics to describe the characteristics of patients diagnosed with TB at participating health facilities and determined the number of TB patients identified in IDRS and TBIMS. We calculated under-reporting as the number of patients diagnosed with TB, but not reported to IDRS or TBIMS after diagnosis. Percent under-reporting was calculated as the number of under-reported TB patients divided by the total number of TB patients diagnosed in the health facility. We described the number and percentage of TB patients by characteristics such as age group, gender, and type of TB. We conducted chi-square tests to assess the association of patient and health facility characteristics with under-reporting. Associations with a *p*-value < 0.2 were included in the multivariable logistic regression model. Crude and adjusted odds ratios (ORs) were calculated to assess odds of under-reporting; 95% confidence intervals of crude OR and adjusted OR, and *p*-value in multivariable logistic regression model were used to evaluate statistical significance of examined characteristics. As EPTB was not mandatorily required to be reported in IDRS, the preliminary results showed an oversized OR value because of “complete separation error” influenced by the variable “Type of TB”. We decided to remove it from the IDRS model after confirming unchanged directions of other variables.

To analyze timeliness and concordance of reporting, we compared the records sampled with corresponding records in health facilities. We defined delayed reporting and recording as the number and percentage of patients diagnosed with TB at the facility but not reported or recorded to IDRS or TBIMS within a day after diagnosis. All analyses were performed in SAS (SAS Institute Inc., Cary, NC, USA).

## 3. Results

In our study, we identified a total of 4698 TB patients (702 from primary health facilities, 1479 from secondary health facilities, and 2517 from tertiary health facilities), of which 4327 were PTB patients (3028 patients diagnosed clinically and 1299 patients confirmed in laboratory) and 371 pleurisy or other EPTB patients. Two thirds (n = 3179) of the patients had national ID recorded. The number of TB patients per county ranged from 320 to 1807. Of all the TB patients, 3658 (77.9%) patients were 15 to 64 years of age, 1000 (21.3%) patients were 65 years or older, and only 40 (0.9%) patients were under 15 years old (Table 1). There were 3127 (66.6%) males and 4117 (87.6%) residents. Most TB patients were selected from the outpatient clinic records (3419; 72.8%), 919 (19.6%) were identified from the inpatient records, and 360 (7.7%) were identified from the laboratory records.

**Figure 1:**
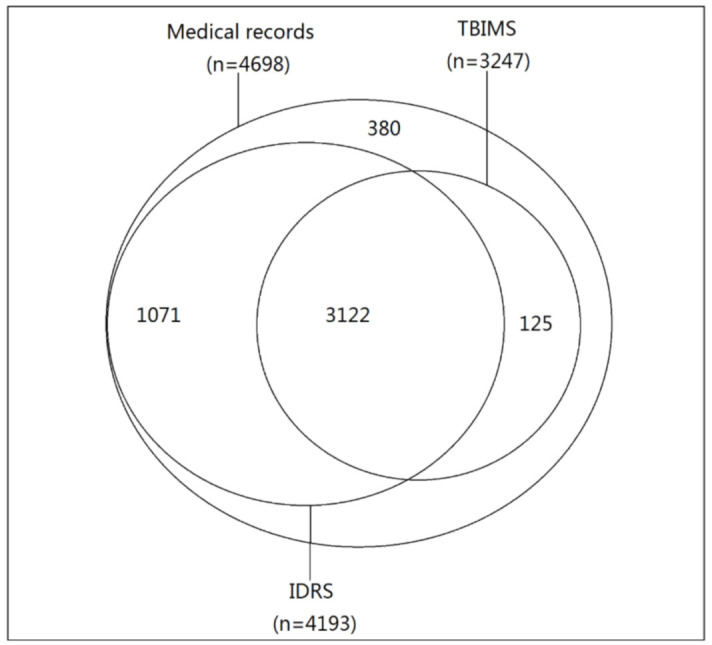
The distribution of TB patients from medical records, IDRS, and TBIMS in the six counties in 2016.

After data matching, we found that 505 (10.8%) patients diagnosed with TB were missing from IDRS, and 1451 (30.9%) patients were missing from TBIMS (Table 2, Figure 1). Compared with the other five counties, Simao county had the largest under-reporting to IDRS and TBIMS (n = 360, 19.9%; n = 919, 50.9%, respectively).

The data source, health facility level, residence, and county were statistically significant factors associated with under-reporting to IDRS in the multivariable analysis (Table 3). The odds of under-reporting to IDRS for a non-resident are 2 times that of a resident. The odds of under-reporting to IDRS for the outpatient data source are about 3 times that of the laboratory data source. The odds of under-reporting to IDRS for tertiary health facilities are about 13 times that of primary health facilities. The risk of under-reporting to IDRS was greatest in Simao, Liyang, and Xingyang counties.

All variables except gender were statistically significant factors associated with under-reporting to TBIMS in the multivariable logistic regression (Table 4). The odds of under-reporting to TBIMS for patients over 65 years of age are almost 2 times that of patients aged 15–64. The odds of under-reporting to TBIMS for clinically diagnosed PTB are about 3 times that of laboratory-confirmed PTB, while the odds of under-reporting for TB pleurisy or other EPTB are 9 times that of laboratory-confirmed PTB. The odds of under-reporting to TBIMS for diagnosis in tertiary health facilities are 10 times that of diagnosis in primary health facilities. The odds of under-reporting to TBIMS for non-residents are 19 times that of residents. The risk of under-reporting to TBIMS is highest in Simao, Nanshan, and Liyang counties. The risk of under-reporting to TBIMS was lower with factors such as outpatients, inpatients, and diagnosis in Xingyang, Yilan, and Lu counties.

In our assessment of concordance and timeliness of reporting to IDRS and TBIMS, 171 records in IDRS and 170 records in TBIMS were included. In IDRS, 18 (10.6%) records of home address and 14 (8.2%) records of diagnosis date were discordant with those in medical records (Table 5). In TBIMS, among demographic variables, the discordant entry of home address was the lowest (12, 7.1%); of all clinical variables, variables about dates, including diagnosis date (5, 2.9%), date of follow-up examination at the end of 2nd month (6, 3.9%), and date of end course examination (22, 14.0%), were the most discordant with medical records. A total of 21 patients (12.3%) were not reported to IDRS and 11 patients (6.5%) were not reported to TBIMS within a day after reporting TB diagnosis.

## 4. Discussion

Our study is an expansion and reinforcement of a previous under-reporting study in China [12]. Both studies explore the possibilities of carrying out the inventory study methodology in China to assess China’s TB surveillance systems. In this study, we performed the assessment in both TBIMS and IDRS and expanded the evaluation involving timeliness, concordance, accuracy, and completeness to conduct a more comprehensive assessment. Additionally, we found and documented some risk factors (high-level hospital and non-resident, etc.) not reported in the previous study.

Our results show that 10.7% and 30.9% of TB patients in the six participating counties were under-reported to IDRS and not recorded for treatment in TBIMS, respectively. The discrepancy in the under-reporting rate between the two systems is mainly because the systems play different roles in the field of public health. Patients diagnosed with PTB in all health facilities should be reported to IDRS, while only patients referred and verified in TB-designated health facilities are recorded in TBIMS. There is value in both vertical (TBIMS) and integrated (IDSR) surveillance systems for capturing data; however, there are potential implications for double reporting or discordance of variables when both surveillance systems are utilized to capture information for the same disease. For example, patients who were misdiagnosed in non-designated health facilities and those who were not traced by TB designated health facilities could be reported to IDRS, but not recorded in TBIMS. This could account for the difference in the under-reporting rate between the two surveillance systems. The under-reporting rate in both IDRS and TBIMS suggests China’s TB surveillance systems may still miss some TB patients. An under-reporting rate to TBIMS of more than one fourth reminds us that there may exist greater risks of under-reporting in certain settings than the WHO estimated national level [8,23]. The under-reported patients, who may not have received proper treatment and support by NTP management, may continue to contribute to TB transmission within their communities.

Our findings are similar to some studies conducted in other countries in which the under-reporting rate of TB ranged from 6% to 68% [14,16,17,24,25,26,27,28,29]. Fatima et al. estimated that almost 68% of persons with TB were not notified to the NTP in Pakistan, in many cases because a large private sector did not report to the NTP [27]. A study conducted in the Republic of Korea estimated that 6% of persons with TB were not reported to the national surveillance system [28]. Our current study shows that the under-reporting rate to TBIMS of the six counties is 30.9%, while a previous study conducted in other areas in China reported the under-reporting rate was 19.3%. The difference between the results of the two studies was mainly because the previous study did not include EPTB except TB pleurisy [12].

Though type of TB was removed from the IDRS model, type of TB was found to be significant for under-reporting to TBIMS. Compared with patients with laboratory-confirmed and clinically diagnosed PTB, patients with TB pleurisy or other EPTB were significantly more likely to be under-reported. This under-reporting associated with type of TB has also been described in other studies [8,12,14,30,31]. Additionally, the reporting requirements of TB pleurisy and other EPTB vary by province. In most provinces, medical institutions are not required to report patients with TB pleurisy. Mandatory reporting of other EPTB is also not required in every province. This is a major challenge and issue; non-standard definitions and reporting requirements severely limit the ability to make comparisons and follow trends.

It is notable from our analysis that the odds of under-reporting are significantly higher in tertiary health facilities compared with primary health facilities. In China, tertiary health facilities are all general hospitals with a high medical level, more than 500 beds, and large numbers of patients being referred to or seeking diagnosis and treatment in these hospitals. Consequently, physicians who are responsible for reporting PTB patients to surveillance systems in tertiary health facilities may spend more time seeing patients than reporting to surveillance systems. Our finding is supported by a study conducted in Kenya, where larger facilities and heavy workload were associated with under-reporting [13]. However, other studies indicated that diagnosis in general hospitals contributes to the notification of TB patients [28,32]. Our findings emphasize the importance of increasing human resource capacity for data collection, management, and reporting of TB patients or shifting these tasks to nurses or non-clinical staff in tertiary health facilities.

Our study revealed substantial under-reporting both to IDRS and TBIMS among non-residents, while the proportion under-reported to TBIMS is much higher. This may be due to economic factors. Most non-resident TB patients are more likely to have low income and lack medical insurance [33]. Non-residents with medical insurance generally received a lower proportion of reimbursement outside of the location in which they are a resident, which may result in refusal of anti-TB treatment. In addition, many non-resident TB patients do not have stable work and a permanent place of residence, which may lead to difficulty in linking the patient to TB services. To strengthen the surveillance of non-resident TB patients, it is necessary to focus on reporting of non-residents and ensure funding is devoted to supporting and following up low-income and non-resident patients. Though information on which facilities had paper records was not collected, facilities that are smaller and located in lower-income areas are more likely (anecdotally) to be paper-based, which could potentially contribute to decreased data quality and reporting.

In our study, under-reporting to TBIMS was also associated with old age. Patients over 65 years old were more likely to be under-reported compared with those aged 15–64 years old. This may be related to a higher rate of comorbidity in patients aged 65 or older, which could necessitate treatment in non-TB-designated health facilities. This is consistent with the bivariate analysis in a study conducted in Spain [14]. We also found that the risk of under-diagnosis was high among patients under 15, which is corroborated by various studies indicated younger age as a risk factor of under-reporting [8,12,32]. However, Kang et al. found that age did not significantly affect TB reporting [28]. The number of TB patients under 15 years old influences study results. In 2016, the WHO estimated that about 11% of TB patients in China were under 15 years old [23]. However, in our study, only 0.9% of TB patients identified were under 15 years old. This may be related to the difference in where children seek healthcare services and the facilities sampled in this study. Most pediatric TB patients are treated in children’s hospitals and therefore reported by regional or national referral hospitals located in big cities. However, none of the hospitals in our selected six counties included this type of hospital. Further studies including children’s hospitals are needed to understand the association between young age and under-reporting of TB patients.

Concordance between systems and reporting of TB patients has been researched in other studies [29,32,34,35,36]. Salyer et al. reported inconsistent smear status of TB patients between records in health facilities and a national surveillance database [29]. Podewils et al. reported a poor agreement of HIV status among TB patients [34]. In our study, address and dates in IDRS and TBIMS were the variables most discordant with medical records. Podewils et al. showed similar findings in their study [34]. This may be due to multiple reasons. Physicians may not have time for adequate epidemiological investigation due to high workload burden in both general hospitals and designated facilities. Patients may also face stigma pressure to not give their private information [37]. Address is important for tracing patients and facilitating full course follow-up, and thus important to include in reporting and recording as an essential variable. Timeliness of reporting was evaluated in multiple studies by different variables, making comparisons difficult. Some studies reported that the proportion of patients with delayed reporting (>7 days) varied from 18.2% to 25.7% [28,32]. Johnson et al. reported that 11% of TB patients were not reported to an electronic communicable disease surveillance system in one business day [38]. Some studies reported the average duration of delay in reporting TB patients (ranging from 4 days to 3 months) [39,40,41]. There was greater under-reporting to TBIMS compared with IDRS. However, when patients were reported, there was greater delay in reporting to IDRS compared with TBIMS (12.3% vs. 6.5%).

This study has demonstrated success in applying TB inventory study methodology in China. Based on this experience, the NTP is planning to integrate routine inventory study activities in the national TB program. The requirements and standard procedure of under-reporting investigation will be formally added in the upcoming newest national TB control guidelines (2021 version). A toolkit will also be issued to train all levels of TB control health workers to implement this work. We expect these activities to help in reducing under-reporting in both systems and improving data recording quality. Moreover, the results and risk factors found can catalyze the TB program and other stakeholders to understand and improve our surveillance systems. The under-reporting and mistakes that occurred in hospitals were often caused by information exchange barriers among different information systems, for example, inconsistency caused by repeat entry, or duplication caused by multiple visits in different hospitals. We aimed to create a platform to enable different systems to exchange patient information, not only data from different departments in the same hospital but also all visits for different diseases in different hospitals. The vision is a Universal Health Care system realized through a unique platform and use of a unique ID (National ID). This can prevent healthcare workers from carrying out unnecessary repeat entry and reduce under-reporting/inconsistency/incompleteness.

Our study has several limitations. First, we used purposive sampling to select six counties, for ease of introducing and implementing matching software; however, this made our findings not representative of other areas in China. Second, we focused on healthcare facilities that had capacity to perform chest x-rays (CXR) and had diagnosed at least one TB patient during the project period, which means patients seeking healthcare in facilities not meeting the criteria were excluded, thus limiting the inclusion of EPTB. It is crucial to ensure that these patients are diagnosed and treated properly. Therefore, under-reporting of under-diagnosed patients deserves further study. Third, children’s hospitals were not included in our study, which could bias our results of under-reporting among children.

## 5. Conclusions

TB patients were under-reported to both China’s national infectious disease reporting system, IDRS, and TB-specific management system, TBIMS. It is important to improve the reporting and recording of TB patients. Local TB programs may focus on reporting and improvement activities in order to support high-patient-load hospitals, non-resident patients, and patients with TB pleurisy or EPTB. Human resource capacity for data collection and management could be enhanced and accuracy of data entry could be reinforced to improve national surveillance systems and TB prevention, diagnosis, and treatment.

## Figures and Tables

**Table 1:** Characteristics of diagnosed tuberculosis (TB) patients in six counties in 2016.

	Diagnosed	
	Patients	%
Total	4698	100
Age		
≤15	40	0.9
15–64	3658	77.9
≥65	1000	21.3
Sex		
Male	3127	66.6
Female	1571	33.4
Type of TB		
PTB clinically diagnosed	3028	64.5
PTB laboratory-confirmed	1299	27.7
Pleurisy or other EPTB	371	7.9
Patient type		
Outpatient	3419	72.8
Inpatient	919	19.6
Lab	360	7.7
Level of health facility		
≤1	702	14.9
2	1479	31.5
3	2517	53.6
Residence		
Resident	4117	87.6
Non-resident	581	12.4
County		
Nanshan	819	17.4
Liyang	379	8.1
Xingyang	441	9.4
Yilan	320	6.8
Lu	932	19.8
Simao	1807	38.5

**Table 2:** The number and percentage of diagnosed TB patients missing from Infectious Disease Reporting System (IDRS) and Tuberculosis Information Management System (TBIMS) in six counties in 2016.

County	Total	IDRS		TBIMS	
		Missing Patients	Proportion (%)	Missing Patients	Proportion (%)
Nanshan	819	50	6.1	283	34.6
Liyang	379	52	13.7	112	29.6
Xingyang	441	33	7.5	43	9.8
Yilan	320	6	1.9	7	2.2
Lu	932	4	0.4	87	9.3
Simao	1807	360	19.9	919	50.9
Total	4698	505	10.8	1451	30.9

**Table 3:** Factors associated with under-reporting to IDRS among diagnosed TB patients in six counties in 2016.

Variables	Total	Under-Reporting		Pearson’s Chi-Square *p*-Value	Crude OR (95%CI)	Adjusted OR (95%CI)	*p-*Value
		N	%				
Total	4698	505	10.7				
Age in years							
<15	40	9	22.5	0.04	2.5 (1.2–5.3)	1.5 (0.6–3.7)	0.4
15–64	3658	383	10.5		ref	ref	
≥65	1000	113	11.3		1.1 (0.9–1.4)	1.3 (1.0–1.6)	0.09
Sex							
Male	3127	301	9.6	0.0005	ref	ref	
Female	1571	204	13.0		1.4 (1.2–1.7)	1.1 (0.9–1.4)	0.4
Data source							
Outpatient	3419	359	10.5	0.06	1.3 (0.9–1.9)	2.7 (1.8–4.1)	<0.001
Inpatient	919	116	12.6		1.6 (1.0–2.4)	0.7 (0.4–1.1)	0.08
Lab	360	30	8.3		ref	ref	
Level of health facility							
≤1	702	14	2.0	<0.0001	ref		
2	1479	110	7.4		4.0 (2.3–6.9)	1.7 (0.8–3.6)	0.1
3	2517	381	15.1		8.8 (5.1–15.1)	12.9 (6.4–25.8)	<0.001
Residence							
Resident	4117	465	11.3	0.0013	ref	ref	
Non-resident	581	40	6.9		0.6 (0.4–0.8)	2.2 (1.2–4.0)	0.009
County							
Nanshan	819	50	6.1	<0.0001	ref	ref	
Liyang	379	52	13.7		2.5 (1.6–3.7)	5.7 (2.8–11.6)	<0.001
Xingyang	441	33	7.5		1.2 (0.8–2.0)	4.5 (2.1–9.8)	<0.001
Yilan	320	6	1.9		0.3 (0.1–0.7)	1.3 (0.4–3.8)	0.6
Lu	932	4	0.4		0.1 (0.0–0.2)	0.0 (0.0–0.1)	<0.001
Simao	1807	360	19.9		3.8 (2.8–5.2)	4.1 (2.2–7.6)	<0.001

**Table 4:** Factors associated with under-reporting to TBIMS among diagnosed TB patients in six counties in 2016.

Variables	Total	Under-Reporting		Pearson’s Chi-Square *p*-Value	Crude OR (95%CI)	Adjusted OR (95%CI)	*p*-Value
		N	%				
Total	4698	1451	30.9				
Age in years							
<15	40	16	40.0	0.0005	1.6 (0.8–3.0)	1.0 (0.5–2.0)	0.9
15–64	3658	1079	29.5		ref	ref	
≥65	1000	356	35.6		1.3 (1.1–1.5)	1.7 (1.4–2.0)	<0.001
Sex							
Male	3127	909	29.07	0.0001	ref	ref	
Female	1571	542	34.50		1.29 (1.13–1.46)	1.04 (0.89–1.21)	0.6
Data source							
Outpatient	3419	807	23.60	<0.0001	0.54 (0.43–0.68)	0.30 (0.21–0.43)	<0.001
Inpatient	919	513	55.82		2.21 (1.72–2.84)	0.54 (0.37–0.79)	0.002
Lab	360	131	36.39		ref	ref	
Type of TB							
PTB clinically diagnosed	3028	984	32.50	<0.0001	1.98 (1.69–2.32)	2.54 (1.94–3.32)	<0.001
PTB laboratory-confirmed	1299	254	19.55		ref	ref	
Pleurisy or other EPTB	371	213	57.41		5.55 (4.33–7.10)	9.27 (6.50–13.23)	<0.001
Level of medical institution							
≤1	702	14	1.99	<0.0001	ref	ref	
2	1479	110	7.44		3.95 (2.25–6.94)	1.77 (0.86–3.65)	0.1
3	2517	381	15.14		8.77 (5.11–15.05)	10.01 (4.99–20.11)	<0.001
Residence							
Local	4117	1147	27.86	<0.0001	ref	ref	
Non-resident	581	304	52.32		2.84 (2.38–3.39)	18.80 (13.77–25.67)	<0.0001
County							
Nanshan	819	283	34.55	<0.0001	ref	ref	
Liyang	379	112	29.55		0.79 (0.61–1.03)	1.56 (1.10–2.20)	0.01
Xingyang	441	43	9.75		0.20 (0.14–0.29)	0.47 (0.31–0.70)	<0.001
Yilan	320	7	2.19		0.04 (0.02–0.09)	0.31 (0.14–0.69)	0.004
Lu	932	87	9.33		0.20 (0.15–0.25)	0.11 (0.08–0.16)	<0.001
Simao	1807	919	50.86		1.96 (1.65–2.33)	5.00 (3.80–6.57)	<0.001

**Table 5:** Concordance of key variables for diagnosed TB patients between facility records and TB surveillance systems (by counties).

Key Variables	Medical Records and IDRS		Medical Records and TBIMS	
	Patients Reviewed in IDRS	Inconsistent with Medical Records	Patients Reviewed in TBIMS	Inconsistent with Medical Records
Total records				
National ID number	161	3 (1.9%)	148	3 (2.0%)
Patient name	171	1 (0.6%)	170	0 (0%)
Address	169	18 (10.7%)	170	12 (7.1%)
Smear status	-	-	170	4 (2.4%)
DST status	-	-	17	0 (0%)
Diagnosis	-	-	170	1 (0.6%)
Treatment outcome	-	-	168	1 (0.6%)
Diagnosis date	171	14 (8.2%)	170	5 (2.9%)
Registration	-	-	170	1 (0.6%)
Date of follow-up examination at the end of 2nd month	-	-	152	6 (3.9%)
Date of end course examination	-	-	157	22 (14.0%)

## Data Availability

All data generated or analyzed during the study are included in this published article. The datasets used and/or analyzed during the present study are available from the corresponding author upon reasonable request.

## Abbreviations

TB	tuberculosis;
WHO	World Health Organization;
PTB	pulmonary TB;
IDRS	Infectious Disease Reporting System;
TBIMS	Tuberculosis Information Management System;
CDC	Center for Disease Control and Prevention;
NTP	national TB program;
PLADs	provincial-level administrative divisions;
CXR	chest x-rays;
EPTB	extra-pulmonary TB;
OR	odds ratio
